# Neuregulin signaling pathway in smoking behavior

**DOI:** 10.1038/tp.2017.183

**Published:** 2017-08-22

**Authors:** R Gupta, B Qaiser, L He, T S Hiekkalinna, A B Zheutlin, S Therman, M Ollikainen, S Ripatti, M Perola, V Salomaa, L Milani, T D Cannon, P A F Madden, T Korhonen, J Kaprio, A Loukola

**Affiliations:** 1 Institute for Molecular Medicine Finland, University of Helsinki, Helsinki, Finland; 2 Broad Institute of MIT and Harvard, Cambridge, MA, USA; 3 Computer Science and Artificial Intelligence Laboratory, MIT, Cambridge, MA, USA; 4 National Institute for Health and Welfare, Helsinki, Finland; 5 Department of Psychology, Yale University, New Haven, CT, USA; 6 Department of Public Health, University of Helsinki, Helsinki, Finland; 7 Estonian Genome Center, University of Tartu, Tartu, Estonia; 8 Department of Psychiatry, Washington University School of Medicine, Saint Louis, MO, USA; 9 Institute of Public Health and Clinical Nutrition, University of Eastern Finland, Kuopio, Finland

## Abstract

Understanding molecular processes that link comorbid traits such as addictions and mental disorders can provide novel therapeutic targets. Neuregulin signaling pathway (NSP) has previously been implicated in schizophrenia, a neurodevelopmental disorder with high comorbidity to smoking. Using a Finnish twin family sample, we have previously detected association between nicotine dependence and *ERBB4* (a neuregulin receptor), and linkage for smoking initiation at the *ERBB4* locus on 2q33. Further, *Neuregulin3* has recently been shown to associate with nicotine withdrawal in a behavioral mouse model. In this study, we scrutinized association and linkage between 15 036 common, low frequency and rare genetic variants in 10 NSP genes and phenotypes encompassing smoking and alcohol use. Using the Finnish twin family sample (*N*=1998 from 740 families), we detected 66 variants (representing 23 LD blocks) significantly associated (false discovery rate *P*<0.05) with smoking initiation, nicotine dependence and nicotine withdrawal. We comprehensively annotated the associated variants using expression (eQTL) and methylation quantitative trait loci (meQTL) analyses in a Finnish population sample. Among the 66 variants, we identified 25 eQTLs (in *NRG1* and *ERBB4*), 22 meQTLs (in *NRG3*, *ERBB4* and *PSENEN*), a missense variant in *NRG1* (rs113317778) and a splicing disruption variant in *ERBB4* (rs13385826). Majority of the QTLs in blood were replicated *in silico* using publicly available databases, with additional QTLs observed in brain. In conclusion, our results support the involvement of NSP in smoking behavior but not in alcohol use and abuse, and disclose functional potential for 56 of the 66 associated single-nucleotide polymorphism.

## Introduction

Smoking is a major risk factor for a variety of somatic diseases and strongly associates with several neuropsychiatric disorders,^1, 2, 3^ the most prominent comorbidity being alcohol use and dependence.^4^ One of the key factors driving persistent smoking is nicotine dependence (ND), manifested by development of tolerance, symptoms of craving and uncontrollable use due to the high addictive potential of nicotine.^5^ Abstaining from smoking results in nicotine withdrawal (NW) symptoms including, for example, irritability, depressed mood and restlessness,^6^ largely contributing to the high relapse rates among smokers trying to quit.^7^ Despite the availability of several smoking cessation pharmacotherapies including nicotine replacement therapy and medications such as varenicline, bupropion and cytisine, 6-month abstinence rates are at best only two- to threefold compared with pharmacologically unassisted quit attempts.^8^

Neuregulin signaling pathway (NSP) is involved in modulating neuronal migration and differentiation. The key functional components of this pathway are neuregulins (*NRG1*, *NRG3*) and their receptor (*ERBB4*), as well as beta secretase *(BACE1)* and the gamma-secretase complex (comprises *PSEN1*, *PSEN2*, *APH1A*, *APH1B*, *PSENEN* and *NCSTN)* (reviewed in ref. 9)). Deviation from an optimal level of NRG/ERBB signaling in the brain is shown to impair brain functions.^10^ Interestingly, several NSP genes have previously been implicated in schizophrenia (SCZ),^9, 10, 11^ a neurodevelopmental disorder with high comorbidity to smoking. Evidence is emerging for the involvement of the NSP in smoking behavior. Recently, we performed a genome-wide association study (GWAS) among smokers from the Finnish Twin Cohort (*N*=1104) and detected association between ND defined by Diagnostic and Statistical Manual of Mental Disorders, 4th edition (DSM-IV)^12^ and *ERBB4.*^13^ Using a partly overlapping subset of families (*N*=505 twins and first degree relatives), we have previously identified linkage between smoking initiation (SI) and microsatellite markers at 2q33, overlapping the *ERBB4* locus.^14^ Further, *Nrg3* has recently been shown to associate with NW in a behavioral mouse model.^15^ Therefore, comprehensive scrutiny of the NSP may aid in identifying a common link for smoking behavior and comorbid disorders.^16^

GWAS has enabled discovery of genetic variants associated with disease traits. However, majority of the identified variants reside in non-coding regions. It is essential to identify the functionality/regulatory potential of the detected variants, for example, via mechanisms such as epigenetic regulation. Functional annotation of variants has boosted progress from genetic studies to biological understanding, aiding in development of therapeutic measures.^17, 18, 19^

In this study, we scrutinized association and linkage between common, low frequency and rare genetic variants in the 10 genes encoding the key functional components of the NSP in a Finnish twin family sample (*N*=1998 from 740 families). To comprehensively test whether NSP variants associate with nicotine use, we included nine smoking-related phenotypes in our analyses. In our secondary analyses, we included five alcohol-related phenotypes to evaluate (i) whether NSP variants associate with nicotine use specifically, or relate to general liability to addictions, and (ii) whether signals observed for smoking-related phenotypes are confounded by comorbid alcohol use. We detected statistically significant association with single-nucleotide polymorphisms (SNPs) and SI, DSM-IV ND and DSM-IV NW, in full agreement with previous studies of NSP in smoking behavior.^13, 14, 15^ Further, we performed comprehensive annotation of the detected associations, and revealed potential functions for majority of the associating SNPs.

## Materials and methods

### Discovery sample

The NAG-FIN discovery sample has been previously described in detail.^13, 14, 20^ Briefly, the study sample was ascertained from the Finnish Twin Cohort consisting of 35 834 adult twins born in 1938–1957. On the basis of earlier data, the twin pairs concordant for ever-smoking were identified and recruited along with their family members (mainly siblings) for the Nicotine Addiction Genetics (NAG) Finland study (*N*=2265). Twin pairs concordant for heavy smoking were primarily targeted to increase the genetic load. Data collection took place in 2001–2005. The association and linkage study sample consisted of 1998 individuals from 740 families, including 980 dizygotic (DZ) twin individuals (both co-twins included), 139 monozygotic (MZ) twin individuals (one co-twin per twin pair was included), 46 individuals with unconfirmed zygosity (due to a lack of DNA sample from the co-twin) and 833 other family members (mostly siblings). The average number of individuals per family was 3 (range 1–10), and the data contained 185 families with at least 4 family members included in the study. Sample description is presented in Table 1. The study was approved by the ethics committee of the hospital district of Helsinki and Uusimaa, Finland in 2001 and 2016, and by the IRB of Washington University, St Louis, MO, USA. The participants have provided written informed consent.

#### NAG-FIN genotype data

Genotyping for the NAG-FIN sample was performed with the Human670-QuadCustom Illumina BeadChip (Illumina, San Diego, CA, USA) (*N*=1097) at the Wellcome Trust Sanger Institute, UK, and with the Illumina Human Core Exome BeadChip (*N*=901) at the Wellcome Trust Sanger Institute, and at the Broad Institute of MIT and Harvard, USA. Quality controls (QC) for the genotype data have been previously described^21^ and are also presented in Supplementary Table 1. Pre-phasing of the data was done with SHAPEIT2^22^ and imputation with IMPUTE2^23^ using the 1000 Genomes Phase I integrated haplotypes reference panel.^24^ For analyses of the 10 NSP genes (*NRG1*,* NRG3*,* ERBB4*,* BACE1*,* PSEN1*,* PSEN2*,* APH1A*,* APH1B*,* PSENEN* and *NCSTN*), we extracted SNPs within the gene regions (according to the longest isoform reported at the UCSC Genome browser) with 50 kb flanking regions. Gene boundaries (according to GRCh37/hg19) are listed in Supplementary Table 1, along with the number of SNPs included for each gene. Only variants with minor allele frequency (MAF) <0.01 located in coding regions, splice sites, promoters or untranslated regions (UTRs) were included in the rare variant analysis. Altogether, 15 036 SNPs were analyzed in our discovery phase.

#### NAG-FIN phenotype data

To evaluate the role of genetic variants within the NSP genes in addictions, we initially tested nine phenotypes encompassing smoking behavior in our discovery sample (Supplementary Table 2 (I)). To test whether variants in NSP genes associate with nicotine use specifically, or relate to general liability to addictions, we also tested five alcohol use phenotypes (Supplementary Table 2 (II)). Data collection for the phenotypes has been previously described in detail.^6, 20^

### Annotation sample

For functional annotation of the associations detected in the discovery phase, we used the DILGOM 2007 (Dietary, Lifestyle and Genetic determinants of Obesity and Metabolic syndrome) study sample, which originates from the population-based national FINRISK 2007 study. The DILGOM study sample has been previously described in detail,^25, 26^ including a total of 631 unrelated Finnish individuals aged 25–74 years from the Helsinki area. For annotation analyses, we used 512 individuals with genome-wide genotype, gene expression and DNA methylation data available, all measured from peripheral blood leukocytes (46% males, mean age 52 years (s.d. 13.7)). DILGOM sample was used to perform population-specific expression (eQTL) and methylation quantitative trait loci (meQTL) analysis in blood tissue. We further analyzed differential transcriptome and methylome among smokers and never smokers in this data set. On the basis of self-reported smoking status the individuals in the sample can be categorized into current daily smokers (*N*=84), current occasional smokers (*N*=34), recent quitters (1–6 months of abstinence) (*N*=13), former smokers (>6 months of abstinence) (*N*=133), never smokers (*N*=245) and three with missing smoking status (as described in ref. 27). The DILGOM participants have provided written informed consent. The protocol was designed and performed according to the principles of the Helsinki Declaration and was approved by the coordinating ethics committee of the hospital district of Helsinki and Uusimaa, Finland.

#### DILGOM genotype data

Genotyping for the DILGOM sample was performed with the Illumina 610-Quad SNP array. Imputation was done with IMPUTE2 using the 1000 Genomes Phase I integrated haplotypes reference panel.^23, 24^ Stringent QC thresholds were applied to filter out low quality SNPs and samples as previously described.^25^ QCs and imputation for all Finnish GWAS data were done centrally at the Institute for Molecular Medicine Finland (FIMM), University of Helsinki, Finland.

#### DILGOM gene-expression data

RNA protocols and data processing for the Illumina HT-12 expression array have been previously described.^25^ Briefly, peripheral blood was used as a source of RNA; all arrays were quantile normalized at the strip-level and technical replicates were combined via bead-count weighted average. Probes were removed if they mapped to a non-autosomal chromosome, erythrocyte globin components or more than one genomic position. A total of 19 probes mapped to the 10 NSP genes, of which 17 passed QC thresholds; none of the *PSENEN* probes passed QC. One probe per gene was selected based on highest interquartile range (IQR) representing highest variance for differential expression analysis.

#### DILGOM DNA methylation data

DNA extracted from peripheral blood was bisulfite converted using EZ-96 DNA Methylation-Gold Kit (Zymo Research, Irvine, CA, USA) according to the manufacturer’s instructions. Illumina Infinium HumanMethylation450 BeadChip was used to measure the DNA methylation levels using the Infinium protocol for methylation workflow.^28^ DNA methylation data was pre-processed and normalized using the bioconductor R package ‘minfi’,^29^ with the Subset-quantile Within Array Normalization (SWAN) method. Probes with detection *P*-value >0.01 in any sample were discarded. Probes reported as cross-reactive (mapping to multiple genomic locations and known repeat regions) and probes with SNPs were also removed, as previously suggested.^29^ A total of 254 CpG probes mapped to the ten NSP genes, of which 226 passed QC thresholds. On average, there were 25 probes per gene (range 13–69).

### *In silico* databases for replicating QTLs identified in blood

We utilized blood-derived gene expression and genotypic data available in the Genotype-Tissue Expression (GTEx) project database (gtexportal.org)^30^ to replicate detected eQTLs in the DILGOM sample. For replicating detected meQTLs in the DILGOM sample, we queried the mQTLdb database (mqtldb.org)^31^ containing blood-derived methylation and genotype data on mother–child pairs.

### *In silico* databases for identifying QTLs in the brain

Given the neuropsychiatric nature of our phenotypes, we tested for eQTLs in different brain tissues using 13 brain tissues available in GTEx and in 10 brain regions available in the Brain eQTL Almanac (BRAINEAC; braineac.org) database.^32^ For meQTLs, we queried the epigenome-wide significant meQTLs reported by Hannon *et al.*^33^ in fetal brain samples (epigenetics.essex.ac.uk/mQTL).

### Schizophrenia twin sample

In the expression analyses of the 10 NSP genes, we also utilized a SCZ twin sample, previously described in detail.^34^ The data consisted of 18 SCZ cases and 55 controls (including unaffected co-twins and additional unaffected twin pairs), with genome-wide gene-expression data generated with Illumina Human WG6 v3.0 Expression BeadChip, as previously described.^34^ One probe per gene was selected in accordance with the probe selection in DILGOM sample for expression analyses. Age and sex was available for all participants, whereas smoking status (defined as smoker versus non-smoker) was available for 13 SCZ cases and 41 controls.

### *In silico* database for SCZ transcriptome analysis

We queried the database for Schizophrenia Genetic Research (SZDB; szdb.org)^35^ to identify differentially expressed NSP genes between SCZ cases and controls.

### Discovery analyses

Altogether, 9924 common (MAF>0.05), 4106 low frequency (0.01⩽MAF⩽0.05) and 1006 rare (MAF<0.01) variants in the ten NSP genes were tested for association initially with nine smoking phenotypes and as secondary analyses with five alcohol-related phenotypes. We also examined common variants for linkage as well as joint linkage and linkage disequilibrium (LD). To account for multiple testing, we used the Benjamini and Hochberg method^36^ and considered false discovery rate (FDR) adjusted *P*-values below 0.05 as statistically significant. To visualize the gene regions showing significant associations in our discovery analyses, we used Locustrack^37^ and Haploview for defining LD blocks based on the ‘solid spine of LD’ option.^38^

#### Genetic association analysis of common and low frequency variants

Univariate linear mixed model (LMM) implemented in GEMMA (genome-wide efficient mixed-model association)^39^ was used to test association of SNPs with the quantitative traits. Genotype uncertainty was accounted for using allelic dosage data. Age and sex were used as covariates (fixed effects part of the model). Population stratification and genetic correlation within the sample was modeled with additional random effects using a standardized relatedness matrix calculated from genome-wide genotype data. *P*-values from Wald test were used to assess the association between each SNP and the phenotype.

#### Linkage and joint linkage and LD analysis of common variants

Two-point linkage analysis as well as joint linkage and LD analysis was performed for binary traits using the PSEUDOMARKER software,^40^ assuming a recessive mode of inheritance (identical to non-parametric affected sib-pair analysis (ASP), which is a special case of parametric linkage analysis).^41, 42^ PSEUDOMARKER has been evidenced as the most powerful family-based association analysis method for binary traits implementing the Elston–Stewart algorithm for full-likelihood analysis.^43^ Only common variants with MAF>0.05 were included in these analyses, to avoid analysis of monomorphic and uninformative markers in the sample. In our linkage analyses, we leveraged the extended twin family data, as our data contained 185 families with at least four family members included in the study.

#### Rare variant association analysis

The rare variant association (RVA) analysis was divided into single-variant and gene-based tests. An expected kinship matrix was calculated using the pedigree information and we incorporated age and sex as covariates. For single-variant RVA analysis of quantitative traits, we used the ‘lmekin’ function from the ‘coxme‘ R package.^44^ To analyze binary traits R package ’pedigreemm’ was employed.^45^ Earlier studies have shown that single-variant tests suffer from loss of statistical power.^46^ Consequently, gene-based tests using SNP-set (Sequence) Kernel Association Test (SKAT)^47^ for quantitative traits and Hierarchical Bayesian Multiple Regression model (HBMR)^48^ for both quantitative and binary traits were performed using R packages. In the gene-based tests, only one variant was selected whenever multiple variants were in full LD, and each variant included had to be at least a singleton (that is, having an imputed genotype>0.5 for at least one individual). HBMR outputs results as Bayes factors (BFs), and we declared a nominally significant finding when BF exceeded a threshold of 2.45 (corresponding to a *P*-value<0.05), as previously suggested.^49, 50^

### Annotations

All SNPs showing statistically significant association or linkage (FDR *P*<0.05) in the discovery analyses were annotated to infer potential functional consequences. For this, we used quantitative trait loci analysis in a population-based sample (blood-derived data), and considered FDR adjusted *P*-values below 0.05 as statistically significant. To replicate QTLs detected in blood, we used publicly available databases. Further, as our phenotypes are neuropsychiatric disorders, we wanted to explore the effect of associating SNPs using publicly available brain tissue data.

#### Expression quantitative trait loci analysis in the DILGOM sample

Expression quantitative trait loci (eQTL) analysis was performed in the DILGOM sample to test the effect of genotypes of the highlighted SNPs on expression levels of NSP genes. R package ‘MatrixEQTL’ was used with a linear model setting.^51^ Age, sex, body mass index (BMI) and smoking status were used as covariates to test *cis*-acting eQTLs. Normalized expression values were log2 transformed and tested against SNP genotypes coded as 0, 1 or 2 copies of the effect allele.

#### Methylation quantitative trait loci analysis in the DILGOM sample

Methylation quantitative trait loci (meQTL) analysis was performed in the DILGOM sample to test the effect of genotypes of the highlighted SNPs on methylation levels of NSP genes. We tested *cis*-acting meQTLs using the R package ‘MatrixEQTL’ with linear model setting. Age, sex, BMI, smoking status and white blood cell counts estimated using houseman algorithm^52^ were added as covariates, as previously suggested.^31, 53^ Normalized methylation *β-*values were tested against SNP genotypes coded as 0, 1 or 2 copies of the effect allele.

#### *In silico* expression quantitative trait loci analysis

To replicate our eQTLs detected in the DILGOM sample (blood), we utilized GTEx whole-blood data. To explore the effects of our highlighted SNPs in the brain, we tested the SNPs as *cis-*eQTLs in 13 brain tissues available in GTEx, and in 10 brain regions available in BRAINEAC. All GTEx analyses (blood and brain tissues) were performed with the ‘Test Your Own’ eQTLs option.

#### *In silico* methylation quantitative trait loci analysis

To replicate our meQTLs detected in the DILGOM sample (blood), we used the mQTLdb assessing *cis*-meQTLs. To test the effect of genotypes in the brain, we queried the fetal brain meQTLs reported by Hannon and colleagues.

#### Other functional annotations

Ensembl Variant Effect Predictor (VEP)^54^ was used to procure predicted functional annotations for the highlighted SNPs. HaploReg v4 was used to annotate the SNPs for regulatory regions (promoter and enhancer histone marks, DNase I hypersensitivity, protein binding and regulatory motifs).^55^ In addition, SPANR^56^ scores for splicing disruption potential were extracted for the highlighted SNPs.

### Differential expression and methylation analyses

As we detected association between smoking behavior and several NSP genes, we wanted to explore whether NSP gene expression and methylation levels are affected by smoking status. We performed differential expression and methylation analysis between never (*N*=245) and current daily smokers (*N*=84) in the DILGOM sample. We also compared never smokers (*N*=245) against a pooled sample of current daily and occasional smokers (*N*=118). We employed linear models using the ‘lm’ function in R while adjusting for age, sex and BMI. When comparing methylation levels, we further adjusted for estimated white blood cell counts.^52^ We considered FDR adjusted *P*<0.05 as evidence for significant difference.

Owing to the high comorbidity between SCZ and smoking, we wanted to explore the effect of smoking on NSP gene expression in the SCZ twin sample. We first examined expression differences between SCZ cases (*N*=18) and controls (*N*=55), while accounting for relatedness and using age and sex as covariates. We then added smoking status as a covariate. In this sample, we could only classify the subjects into smokers and non-smokers, as there was no information on former smoking. Owing to the small sample size, no correction for multiple testing was applied.

## Results

### Discovery analyses

We tested 15 036 SNPs mapping to the 10 NSP genes (with 50 kb flanking regions) for linkage, joint linkage and LD, and association identifying altogether 66 SNPs showing statistically significant signal (FDR *P*<0.05) with SI, ND and NW phenotypes (Supplementary Table 3). On the basis of LD structures, the 66 SNPs represent 23 LD blocks (Supplementary Figure 1). A summary of the results from the discovery analyses is presented in Table 2.

We detected evidence of linkage for SI, ND diagnosis and NW diagnosis with multiple SNPs in *ERRB4*, with the strongest signal emerging in rs1836721 for SI (log of the odds (LOD)=3.32) and ND (LOD=1.94), and in rs147786642 for NW (LOD=1.12).

Joint linkage and LD tests performed with PSEUDOMARKER detected a signal for SI in seven of the ten NSP genes (*APH1A*, *PSEN2*, *ERBB4*, *NRG1*, *NRG3*, *BACE1* and *PSENEN*; FDR *P*<0.05) (Supplementary Figure 2), and signal for ND diagnosis in *ERBB4* (FDR *P*=0.002 for rs13385826) (Supplementary Figure 3).

**Figure 1:**
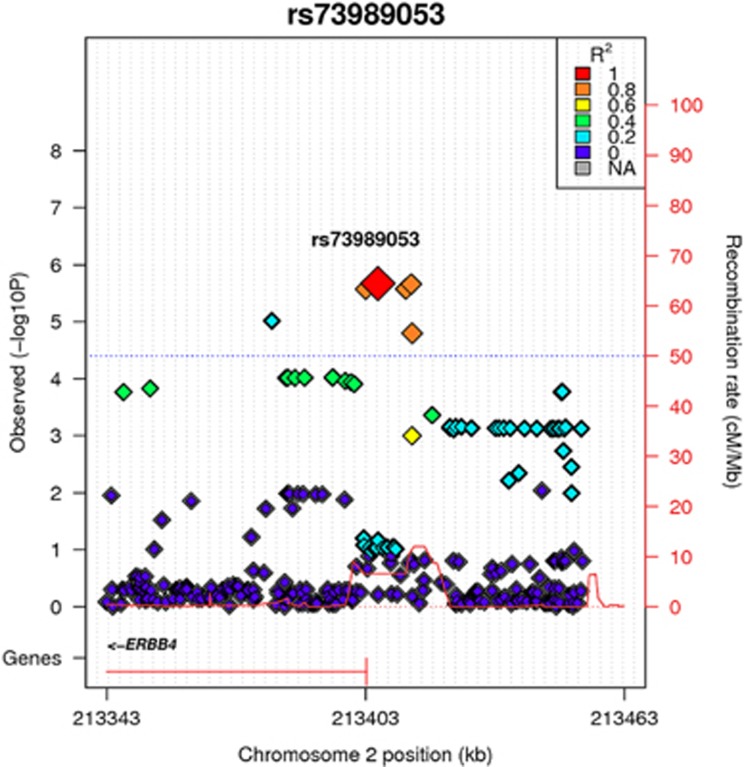
Regional plot for rs11192578 in *ERBB4* gene showing association with nicotine withdrawal (NW) symptom count.

Association tests performed with GEMMA identified a signal for NW symptom count in *ERBB4* (FDR *P*=0.008, *β*=−0.517 for rs13001305, and for three highly correlated SNPs: rs73989053, rs13006797, rs17328083) (Figure 1), suggesting that each copy of the effect allele lowers number of NW symptoms by half a count on a scale of 0–8 counts. Similar results were obtained for *NRG3* SNP rs11192578 (FDR *P*=0.008, *β*=−0.773) (Supplementary Table 3), suggesting that each copy of the effect allele lowers number of NW symptoms by 0.8 counts.

No significant signal was detected in single-variant RVA analyses after correcting for multiple testing (data not shown). In the gene-based RVA analysis, neither methods (SKAT or HBMR) detected significantly associated variants for ND or NW symptom counts. In contrast, HBMR identified association between SI and both *NRG1* (BF=3.073, *P*<0.0360) and *PSEN1* (BF=9.693, *P*<0.0078), and between NW diagnosis and *ERBB4* (BF=3.799, *P*<0.0267).

### Annotation

To test whether the 66 SNPs highlighted in the discovery phase associated with gene expression or DNA methylation levels of the NSP genes, we performed *cis* eQTL and meQTL analyses in the DILGOM sample (blood-derived data). Out of the 66 SNPs, two SNPs in *ERBB4* and 23 SNPs in *NRG1* were identified as eQTLs (FDR *P*<0.05) (Supplementary Table 4). Further, 12 SNPs in *ERBB4* (eight of those forming meQTLs with multiple CpG sites), one SNP in *PSENEN*, and one SNP in *NRG3* (FDR *P*<0.05) were identified as altogether 22 meQTLs (Supplementary Table 5).

To replicate our eQTL findings, we tested the 66 highlighted SNPs for *cis*-eQTLs in whole-blood data available in GTEx. We observed 17 eQTLs in *NRG1*, overlapping the 23 *NRG1* eQTLs identified in DILGOM sample, and additional eQTLs in *BACE1* and *PSENEN* (FDR *P*<0.05) (Supplementary Table 6). We then tested all 66 SNPs for *cis*-eQTLs in RNA expression data from 13 brain regions available in the GTEx and 10 brain regions available in the BRAINEAC database. Altogether, five brain eQTLs were detected: one *ERBB4* SNP (rs13385826) in spinal cord, one *NRG3* SNP (rs12774918) in amygdala, one *BACE1* SNP (rs1261780) in cerebellar hemisphere and two *ERBB4* SNPs (rs192584214, rs112465988) in cerebellar cortex (FDR *P*<0.05) (Supplementary Table 7). Overlap between eQTLs detected in brain and blood (GTEx) was observed only for rs1261780 in *BACE1*.

To replicate our meQTLs findings, we tested the 66 highlighted SNPs for *cis-*meQTLs in mQTLdb. Twelve meQTLs identified in DILGOM sample overlapped with results from mQTLdb with additional meQTLs observed for rs807483 in *PSENEN* (Supplementary Table 8). In addition, among the epigenome-wide significant meQTLs reported in fetal brain by Hannon and colleagues, we found four of the *ERBB4* SNPs (rs73989053, rs17328083, rs13001305 and rs75489550) (Supplementary Table 9), all overlapping with meQTLs in the DILGOM sample, and all showing association with NW in our discovery sample.

Among the highlighted 66 SNPs, Ensembl Variant Effect Predictor (VEP) indicated a missense variant in *NRG1* (rs113317778), promoter region variants in *NRG1* (rs75673683) and *ERBB4* (rs73989053), and two *APH1A* SNPs (rs183423866, rs187135585) that overlap with transcription factor binding sites (Supplementary Table 10). According to HaploReg, a significant portion of *ERBB4* and *NRG3* SNPs overlap with promoter and/or enhancers in brain tissue (Supplementary Table 11).

Most of the highlighted 66 SNPs were either too far from splice junctions or had no overlapping coding transcripts for splicing disruption analysis with SPANR. Only *ERBB4* variant rs13385826 had a delta psi score of 0.09 indicating splicing disruption potential (absolute delta psi>0.05).

**Figure 2:**
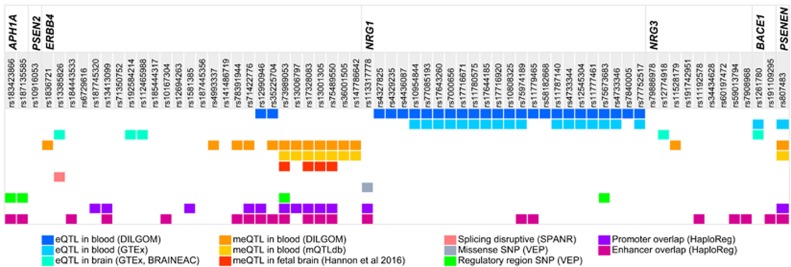
Summary figure depicting the functional annotation of the 66 single-nucleotide polymorphisms (SNPs) highlighted in the discovery phase. The figure presents the summary of functional annotation results for the 66 SNPs highlighted in the discovery analyses while showing the overlap between multiple functional annotations for each SNP. In addition to the eQTLs and meQTLs identified in blood and brain, we identified one splicing disruption variant (rs13385826) with SPANR, one missense variant (rs113317778), and four variants in regulatory regions (promoter (and flanking) region and transcription factor binding sites) with some SNPs overlapping promoter and enhancer in blood and brain.

Annotation results for the 66 SNPs are summarized in Figure 2 and in Supplementary Table 3.

### Differential expression and methylation analyses

When examining the expression of the ten genes, we observed higher expression levels of *NRG1* and *PSEN1* among current daily versus never smokers (FDR *P*<0.05) (Supplementary Table 12). When occasional smokers were pooled together with current daily smokers, the differences were no longer significant. Expression levels in occasional smokers resembled those seen in never smokers (Supplementary Figure 5). No significant difference in methylation levels between current daily versus never smokers were observed (Supplementary Table 13), including occasional smokers among current daily smokers did not affect this result.

In the SCZ twin sample, *NRG1*, *NRG3* and *APH1B* showed a trend (*P*-value<0.1) for differential expression between SCZ cases and controls in analyses adjusted for age and sex (Supplementary Table 14). When smoking status was added to the model, the trend disappeared.

## Discussion

Smoking behavior and ND are complex traits with several genes and pathways having crucial roles. In addition to the well-established role of genetic variation in nicotinic receptor genes^57, 58, 59^ and nicotine metabolizing enzymes,^60, 61, 62^ the NSP is emerging as a contributor in smoking behavior. By using a Finnish twin family sample, we have previously identified linkage of SI at the *ERBB4* locus,^14^ and association between ND and *ERBB4.*^13^ Turner *et al.*^15^ have recently shown the involvement of *Nrg3* and *Erbb4* in the anxiety effects of NW in a behavioral mouse model, and association between *NRG3* SNPs with smoking cessation success in a clinical trial. In the current study, we included 14 phenotypes assessing smoking behavior and alcohol use in a Finnish twin family sample (*N*=1998 individuals from 740 families). We scrutinized altogether 15 036 common, low frequency and rare variants in 10 NSP genes, and identified 66 SNPs (representing 23 LD blocks) significantly associated with SI, ND and NW. We then comprehensively annotated the functional potential of the highlighted variants in an independent population-based sample of smokers and non-smokers.

On the basis of LD figures generated for the seven genes, we estimate that the 66 highlighted SNPs represent 23 LD blocks. For all but three genes (*APH1A*, *PSEN2* and *PSENEN*), SNPs in multiple LD blocks were highlighted. We detected linkage for SI with multiple SNPs in *ERBB4*, strengthening our previous linkage findings.^14^ Our sample size increased between the original linkage study and the current study (*N*=505 versus *N*=1998), and the evidence for linkage increased correspondingly (max parametric LOD=2.56 versus max LOD=3.32), despite the fact that biallelic markers (that is, SNPs) used in the current study provide less linkage information individually compared to multiallelic markers (that is, microsatellite markers)^63^ used in the original linkage study.^14^ *ERBB4* variants also showed linkage for ND diagnosis (LOD=1.94) and NW diagnosis (LOD=1.12), joint linkage and LD for SI and ND diagnosis, and association for NW symptom count. These findings further support our previous association results^13^ and provide substantive evidence for future studies to characterize the role of *ERBB4* in addictions.

In addition to highlighting *ERBB4*, our results provide evidence for the involvement of other NSP genes in smoking behavior. A SNP in *NRG3* was associated with NW symptom count, in line with findings from animal models and association analyses reported by Turner *et al.*^15^ The effect sizes detected for *ERBB4* and *NRG3* SNPs showing association with NW symptom count are prominent, corresponding to a decrease of 0.5–0.8 counts on a scale of 0–8 counts, per each copy of the effect allele. Further, joint linkage and LD analysis detected a signal for SI in seven of the NSP genes (majority of the highlighted SNPs), likely due to our discovery sample being heavily enriched for smoking (83% having initiated smoking) providing ample statistical power in our family sample. In our rare variant gene-based analyses using HMBR, we observed significant association of *ERBB4*, *NRG1* and *PSEN1* with SI and NW diagnosis. Overall, we provide strong evidence implying the NSP in smoking behavior while highlighting novel associations in five genes (*NRG1*, *BACE1*, *APH1A*, *PSEN2* and *PSENEN*) for smoking behavior. It is noteworthy that among the 14 tested phenotypes (nine assessing smoking behavior and five alcohol use), the signal consistently emerges for (i) moking initiation, supporting our previous linkage finding for smoking initiation on 2q33, overlapping the *ERBB4* locus,^14^ (ii) DSM-IV ND, supporting our previously reported association between *ERBB4* and DSM-IV ND^13^ and (iii) NW, supporting previous findings by Turner *et al.*^15^ No significant signal was detected for ND assessed by FTND or smoking quantity. Further, no signal was detected for alcohol use phenotypes, suggesting that the role of the NSP is specific to nicotine, rather than being broadly involved in addictions in general. Lack of signal for alcohol use phenotypes also suggests that signals observed for smoking-related phenotypes are not confounded by comorbid alcohol use. Future studies are needed to assess the involvement of the NSP in use and dependence of other substances besides nicotine and alcohol. In our discovery data set, cannabis or other illicit drug use is very rare.^64^

Altogether, 38 of the 66 highlighted SNPs were identified either as eQTLs or meQTLs in the DILGOM sample, suggesting that they may affect expression and methylation levels of the NSP genes. Also, five of the 66 SNPs were detected as eQTLs in different brain tissues (cerebellar cortex, cerebellar hemisphere, amygdala and spinal cord) and four as meQTLs in fetal brain, indicating that at least some of our highlighted SNPs may affect gene expression or methylation in the brain. Three brain regions (amygdala, cerebellar cortex and cerebellar hemisphere) showing eQTLs in our study have previously been implicated in addiction studies.^65, 66, 67, 68^ Although spinal cord, another tissue showing eQTLs in our study, has not been studied previously with respect to addictions, our findings from GTEx data suggest spinal cord should be considered in addiction studies.^30^

Smoking has a prominent effect on methylation,^69^ and alters gene expression.^70^ Unfortunately, most publicly available eQTL and meQTL databases do not currently provide information on smoking status. Nevertheless, we could replicate most eQTLs and meQTLs detected in the DILGOM sample in blood. For example, out of the 23 *NRG1* eQTLs detected in the DILGOM sample (blood), 17 replicated in GTEx (blood) but none were observed in brain tissue (GTEx, BRAINEAC), however, among the 20 *ERBB4* meQTLs detected in the DILGOM sample (blood), 17 replicated in mQTLdb data (blood) and 4 of were also observed in fetal brain. Such inconsistent overlap may stem from differences in gene expression and methylation levels across tissues. Additional factors contributing towards incomplete overlap of QTL results between blood-derived results in DILGOM versus public databases (GTEx and mQTLdb) may include population-specific genetic background and lack of detailed phenotypic information (like smoking status) available in the databases. Further, small sample sizes for tissues that are difficult to access, such as brain, reduce the power to identify signals. In addition, data made available from published studies often only contain (epi)genome-wide significant results thereby limiting the possibility of replicating results of targeted studies like ours. Nonetheless, epigenome-wide significant meQTLs reported for the highlighted 66 SNPs in fetal brain overlap with meQTLs observed in DILGOM sample (blood data), indicating very robust associations between SNPs and methylation levels of these genes. The four *ERBB4* promoter region meQTLs detected in both blood and fetal brain showed association with NW. These meQTLs were not detected in adult brain tissues in the same study by Hannon *et al.*;^33^ as smoking was not accounted for in this study, gene-by-environment interactions induced by smoking exposure cannot be ruled out. If future studies reveal that methylation at these CpG sites react to smoking exposure, this locus could be a potential target for epigenetic therapy for NW.

Owing to the limited access and availability of brain tissue, the use of blood as a substitute for transcript level analyses has been evaluated, and a modest overlap of ~19% has been observed, emphasizing the value of tissues that are specific to the pathophysiology of the trait of interest.^71, 72^ However, blood can be used as surrogate for brain tissues, especially for genes that are co-expressed between the two tissues.^73^ According to GTEx, seven out of the ten NSP genes are co-expressed in brain and blood. However, the expression levels differ significantly for almost all the genes between brain and blood.

Alternative splicing of *ERBB4* has been reported to result in functionally distinct isoforms that can alter downstream signaling.^74^ Interestingly, the ND-associated SNP rs13385826 in *ERBB4* is predicted to be a splicing disruption variant according to SPANR, plausibly inducing alternative splicing of exon 21 encoding for part of the kinase domain of *ERBB4*, crucial for downstream signaling.^75^ Functional validation of the splicing product of rs13385826 is needed to confirm the specific *ERBB4* isoform and its consequences to the properties of the protein. Downstream targets of the NSP are being evaluated as drug targets in SCZ.^76, 77^ Despite only one SNP being in the coding region, there appears to be regulatory potential in 56 of the highlighted 66 SNPs by means of eQTLs, meQTLs and other regulatory features (overlapping enhancer and/or promoter or transcription factor binding sites) (Figure 2). However, further validation studies are required to confirm the predicted function of these SNPs.

In our expression analyses, we observed differential expression of *NRG1* and *PSEN1* between current daily and never smokers in the DILGOM sample, suggesting that smoking influences the expression of at least some of the NSP genes. Interestingly, merging current daily and occasional smokers diluted the signal. Further, our data show that expression levels of occasional smokers resemble more never smokers compared with the daily smokers. Occasional smokers by definition smoke irregularly and hence alternate between exposure and non-exposure to tobacco and its chemicals. This underlies the importance of careful definition of smoking status in gene-expression studies.

Differential expression in *NRG1*, *NRG3*, *PSEN1*, *PSEN2*, *PSENEN* and *APH1A* (FDR *P*<0.05) has been reported between SCZ cases and controls in SZDB;^35^ strikingly, smoking status has not been accounted for in any of the studies included in this database. In the current study, we detected a trend for differential expression of *NRG1*, *NRG3* and *APH1B* in our small SCZ twin sample (*N*=73). However, after adjusting for smoking status the trend disappeared. Given the high co-occurrence and shared genetic component of SCZ and smoking,^78^ and our findings, the results from SZDB may partly reflect smoking status rather than the disease status, advocating the importance of accounting for confounding effects of smoking.

We detected no significant differences in NSP gene methylation levels between current daily and never smokers in the DILGOM sample. This suggests that the microarray used in this study may not have covered relevant CpG sites, or that other regulatory mechanisms besides methylation account for the detected differences in expression levels. Pooling occasional smokers together with current daily smokers had no effect, suggesting that methylation of NSP genes does not change rapidly in response to abstinence.

In our analyses, we utilized a Finnish twin family sample, heavily enriched for smoking. As the participants were on average 56 years old at the time of enrollment, we were not able to recruit parents but rather took advantage of the large number of affected sib-ships. We applied diligent quality controls to exclude low quality SNPs. Although the widely used threshold of imputation info score of >0.4 was applied, the average info score of the 66 highlighted SNPs was 0.89 (median 0.93), and only two SNPs had an info score <0.7. *NRG3* SNP rs11192578 showed mild LD with neighboring SNPs plausibly explained by suggestive evidence of positive selection (iHS score 1.7 obtained from Haplotter (haplotter.uchicago.edu)^79^ and low minor allele frequency (MAF=0.04) in our sample. *ERBB4* SNP rs13385826 (showing association with ND diagnosis) appears to be an orphan signal with no support from surrounding SNPs despite having several SNPs in high LD.

In conclusion, our twin family sample provided further evidence for the involvement of the NSP in smoking behavior but not in alcohol use and abuse phenotypes. Our differential expression analyses highlighted the importance of carefully defining the smoking status in gene-expression studies. Our results underlined the involvement of *ERBB4* in SI, ND and NW. Further, we highlighted *NRG3* for SI and NW, and additional five NSP genes for SI. By using both in-house data and publicly available databases we depicted potential function for an exceptionally high proportion (56/66) of the associated SNPs, suggesting the potential of these variants as future drug targets.

## Figures and Tables

**Table 1:** Discovery sample characteristics

*Sample characteristics*	*Descriptive statistics*
Total sample size (% males)	1998 (52%)
Number of families	740
Mean age (range, s.d.)	56 (30–92, 10.1)
Fulfilling smoking initiation^a^ (%)	1660 (83%)
Mean age at smoking initiation (range, s.d.)	18.5 (7–56, 4.96)
Mean CPD (range, s.d.)^b^	18.8 (1.5–45, 10.2)
Mean DSM-IV ND symptoms (range, s.d.)^b^	2.9 (0–7, 1.7)
Fulfilling DSM-IV ND diagnosis (%)^b^	844 (42%)
Mean DSM-IV NW symptoms (range, s.d.)^c^	2.3 (0–8, 2.1)
Fulfilling DSM-IV NW diagnosis^c^	522 (26%)

Abbreviations: ND, nicotine dependence; NW, nicotine withdrawal.

a Smoked ⩾100 cigarettes during lifetime.

b Among those who have initiated smoking.

c Among those who have initiated smoking, and have attempted quitting.

**Table 2:** Summary of statistically significant (FDR *P*<0.05) results from discovery phase

*Gene symbol*	*Gene name*	*Chr*	*No. of SNPs highlighted*	*Phenotypes showing association*	*SNPs with lowest *P*-value*	
					*rs number*	P*-value*
*APH1A*	Aph-1 homolog A	1	2	Smoking Initiation	rs183423866	1.8E−09
*PSEN2*	Presenilin 2	1	1	Smoking Initiation	rs10916053	2.1E−04
*ERBB4*	Erb-B2 Receptor Tyrosine Kinase 4	2	27	Smoking Initiation	rs13413099	4.0E−27
				Nicotine Depdendence	rs13385826	1.7E−07
				Nicotine Withdrawal	rs13006797	2.1E−06
*NRG1*	Neuregulin 1	8	24	Smoking Initiation	rs4329235	9.3E−13
*NRG3*	Neuregulin3	10	9	Smoking Initiation	rs11528179	2.9E−07
				Nicotine Withdrawal	rs11192578	1.2E−06
*BACE1*	Beta-secretase 1	11	2	Smoking Initiation	rs191109295	4.0E−06
*PSENEN*	Presenilin enhancer	19	1	Smoking Initiation	rs807483	1.7E−04

Abbreviations: FDR, false discovery rate; ND, nicotine dependence; NSP, neuregulin signaling pathway; NW, nicotine withdrawal; SI, smoking initiation; SNP, single nucleotide polymorphism. Altogether, 66 SNPs were highlighted in seven of the ten NSP genes with SI, DSM-IV ND diagnosis and DSM-IV NW symptom count phenotypes. For each gene, SNP with the lowest *P*-values are presented. Complete results for 66 SNPs are presented in Supplementary Table 3.
