# Supplementary material for: Neuregulin signaling pathway in smoking behavior
Source: Transl Psychiatry. 2017 Aug 22;7(8):e1212–. doi: 10.1038/tp.2017.183 (PMC5611747; doi:10.1038/tp.2017.183)

Supplementary figure 1. Haploview plots showing the LD structure for the highlighted 66 SNPs in *NRG1, ERBB4, NRG3, BACE1, APH1A,* and *PSEN2* in discovery phase. For each gene plots indicating R^2 and D' values are presented. Block boundaries were defined by the 'Solid spine of LD' option of Haploview.

a) NRG1

R^2

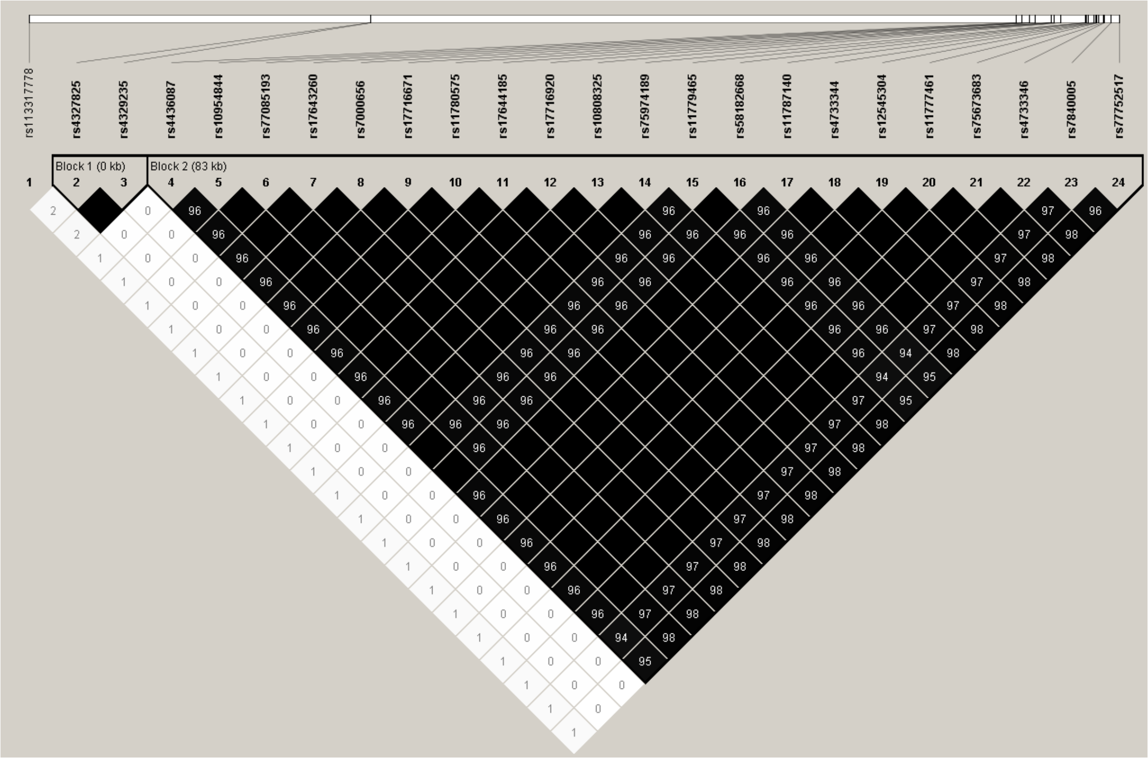

D'

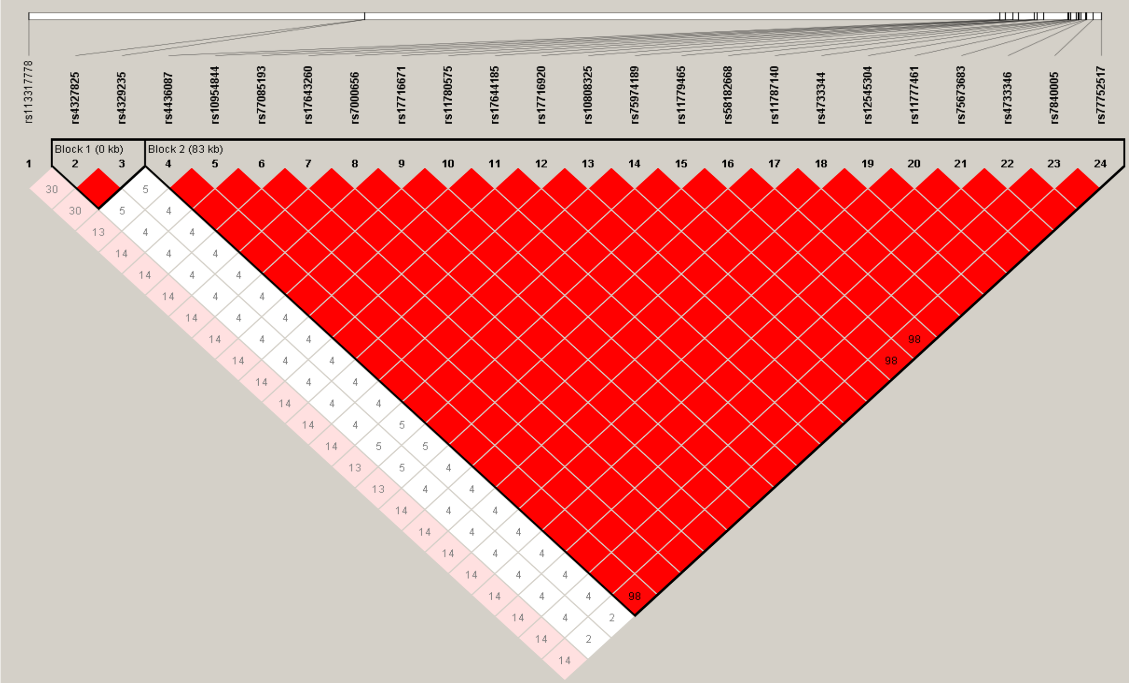

b) *ERBB4*

R^2

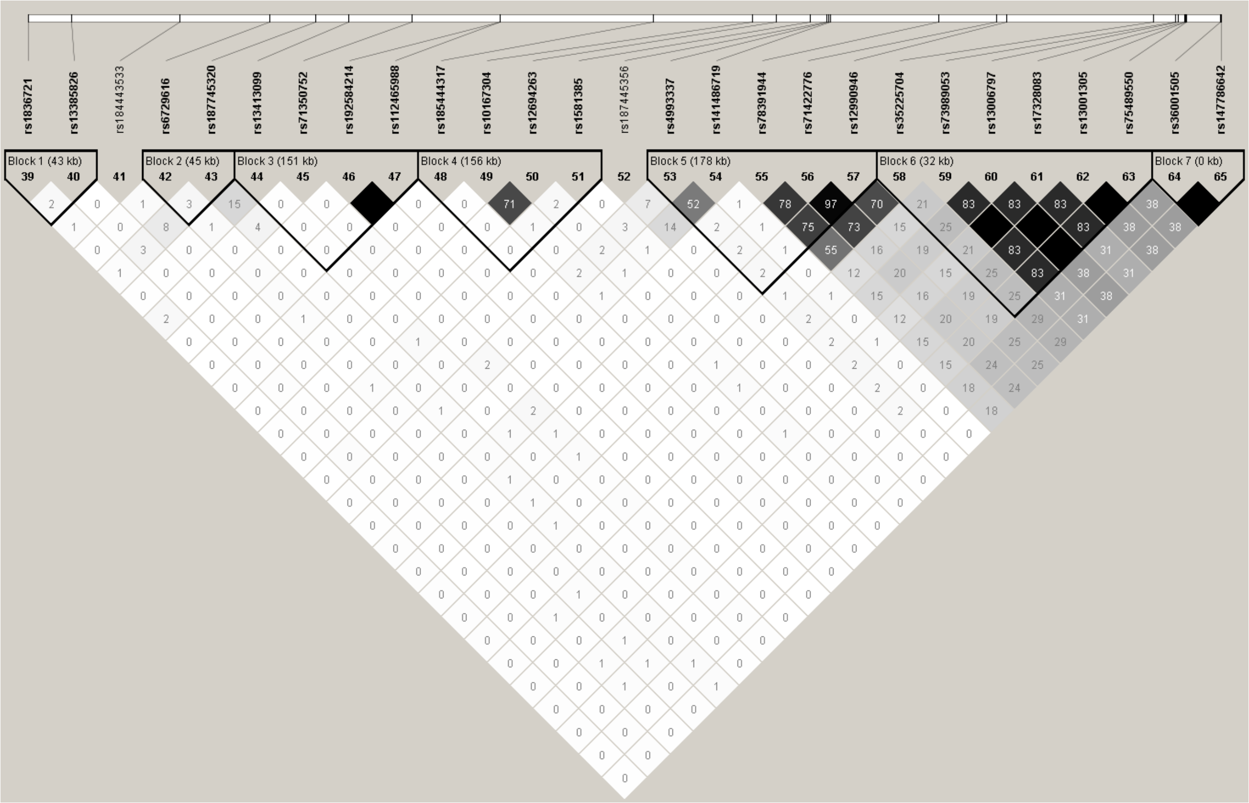
rs1836721
rs13385826
rs184443533
rs6729616
rs187745320
rs13413099
rs71350752
rs192584214
rs112465988
rs185444317
rs10167304
rs12694263
rs1581385
rs187445356
rs4993337
rs141486719
rs78391944
rs71422776
rs12990946
rs35225704
rs73989053
rs13006797
rs17328083
rs13001305
rs75489550
rs36001505
rs147786642
Block 1 (43 kb)
Block 2 (45 kb)
Block 3 (151 kb)
Block 4 (156 kb)
Block 5 (178 kb)
Block 6 (32 kb)
Block 7 (0 kb)

D’

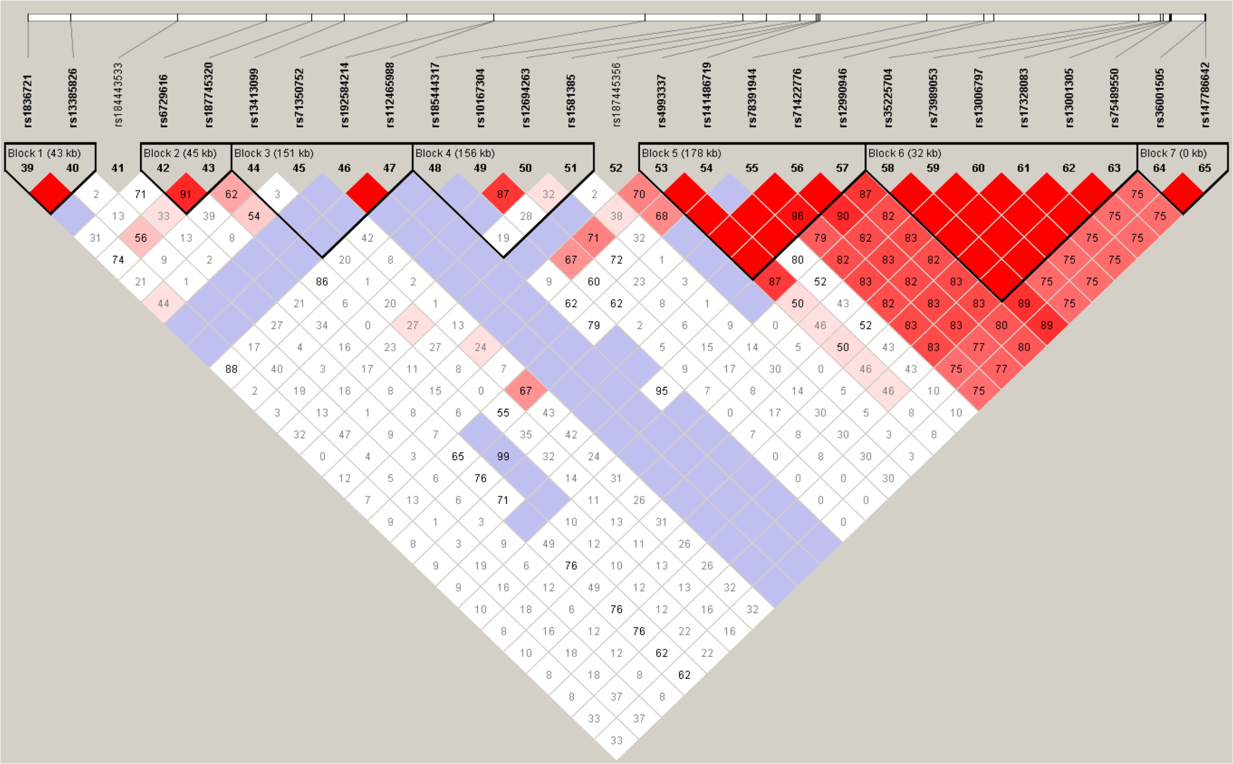
rs1836721
rs13385826
rs184443533
rs6729616
rs187745320
rs13413099
rs71350752
rs192584214
rs112465988
rs185444317
rs10167304
rs12694263
rs1581385
rs187445356
rs4993337
rs141486719
rs78391944
rs71422776
rs12990946
rs35225704
rs73989053
rs13006797
rs17328083
rs13001305
rs75489550
rs36001505
rs147786642
Block 1 (43 kb)
Block 2 (45 kb)
Block 3 (151 kb)
Block 4 (156 kb)
Block 5 (178 kb)
Block 6 (32 kb)
Block 7 (0 kb)

c) *NRG3*

R^2

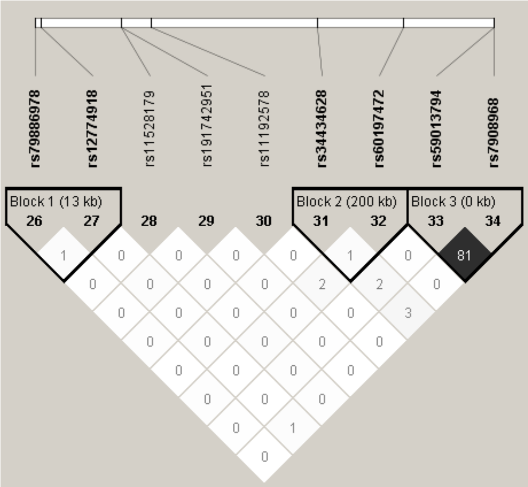

D’

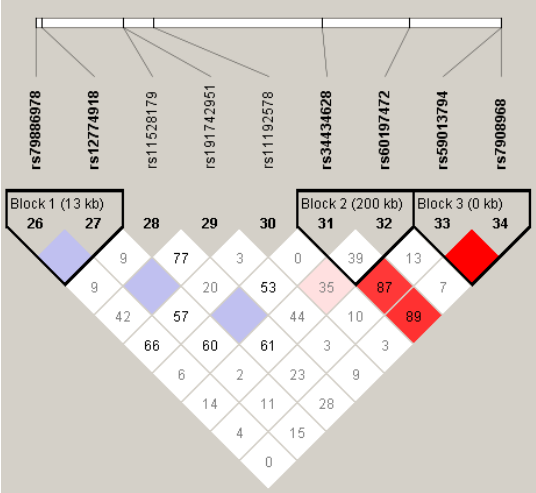

d) *APH1A* and *PSEN2*

R^2

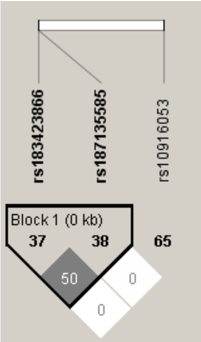

D’

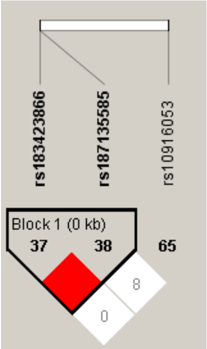

e) *BACE1*

R^2

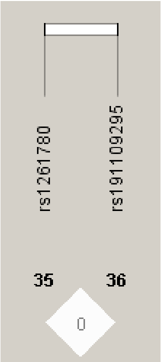

D’

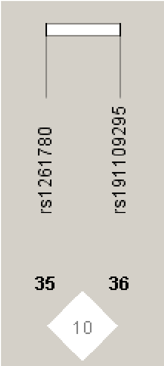

Supplement: Supplementary Figure 1 [file tp2017183x1.pdf]
