# Supplementary material for: Neuregulin signaling pathway in smoking behavior
Source: Transl Psychiatry. 2017 Aug 22;7(8):e1212–. doi: 10.1038/tp.2017.183 (PMC5611747; doi:10.1038/tp.2017.183)

Supplementary figure 2. Regional plots for the SI associated SNPs in *ERBB4, NRG1, NRG3, BACE1, APH1A, PSEN2,* and *PSENEN*. Threshold line corresponds to FDR p=0.05.

ERBB4

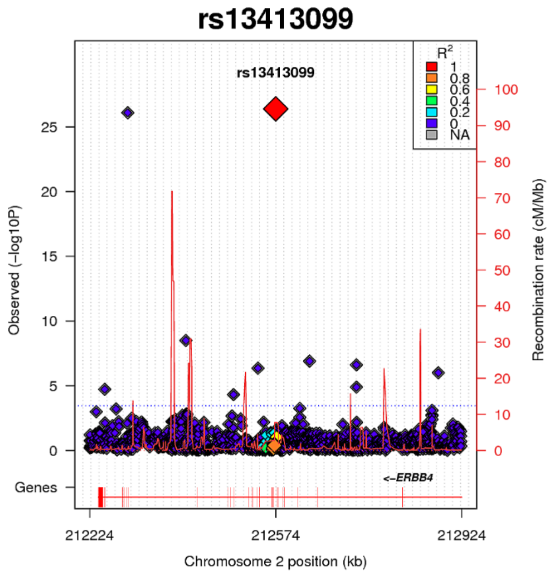

NRG1

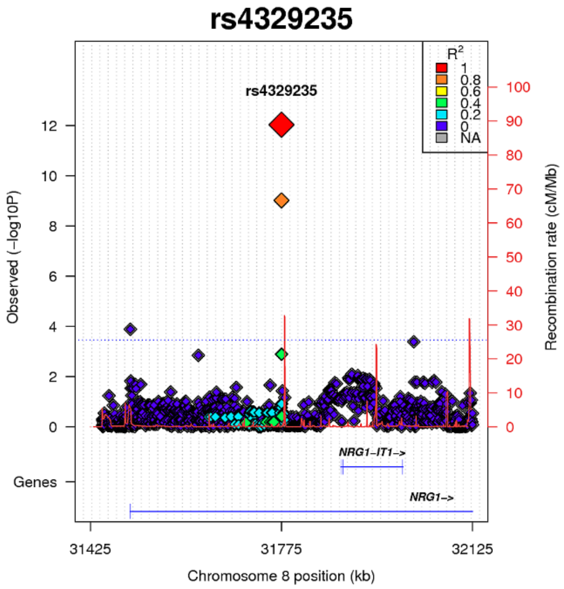

NRG3

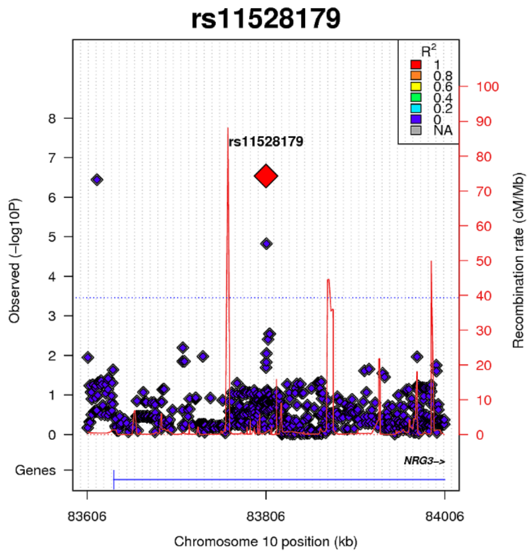

BACE1

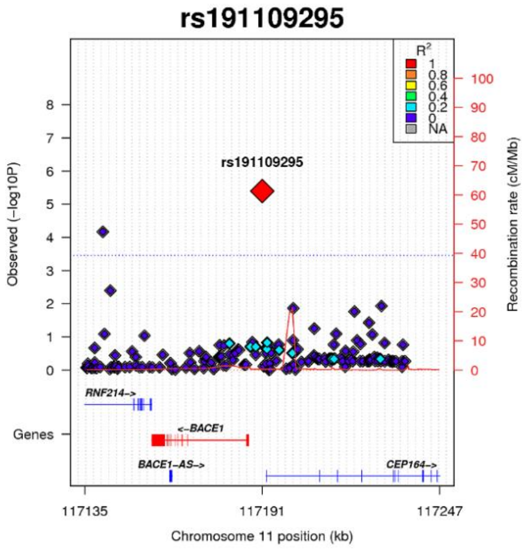

APH1A

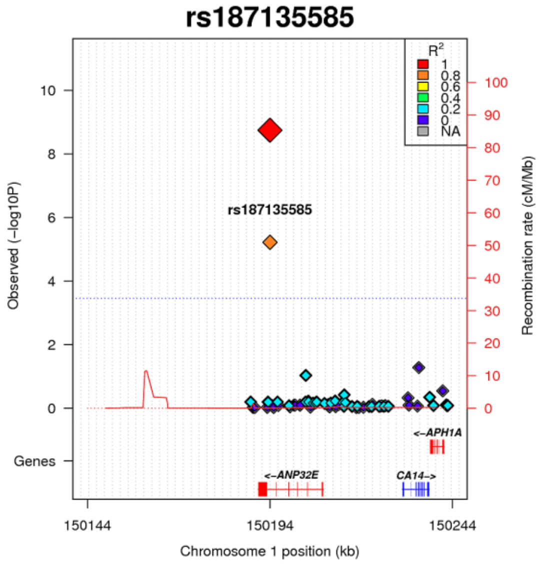

PSEN2

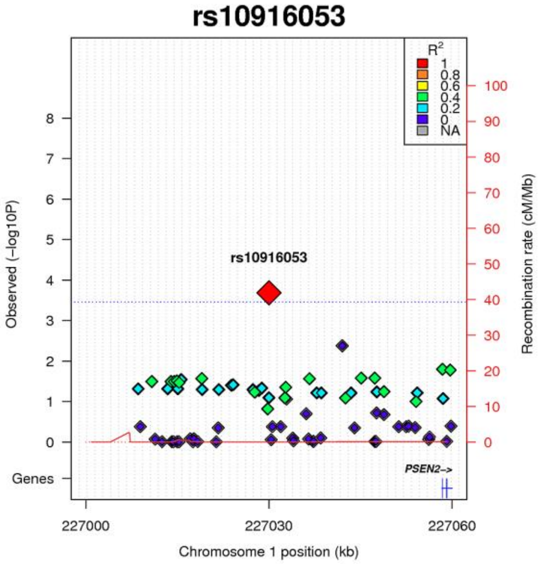

PSENEN

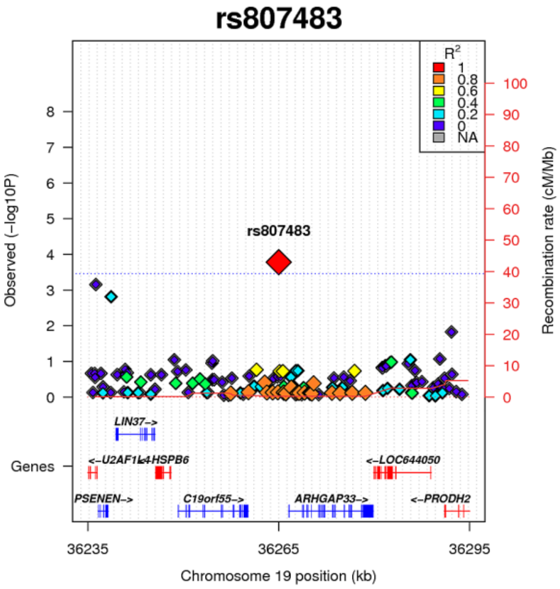

Supplement: Supplementary Figure 2 [file tp2017183x2.pdf]
