# Supplementary material for: Neuregulin signaling pathway in smoking behavior
Source: Transl Psychiatry. 2017 Aug 22;7(8):e1212–. doi: 10.1038/tp.2017.183 (PMC5611747; doi:10.1038/tp.2017.183)

Supplementary figure 3. Regional plot for *ERBB4* SNP rs13385826 showing association with ND diagnosis.

Threshold line corresponds to FDR p=0.05.

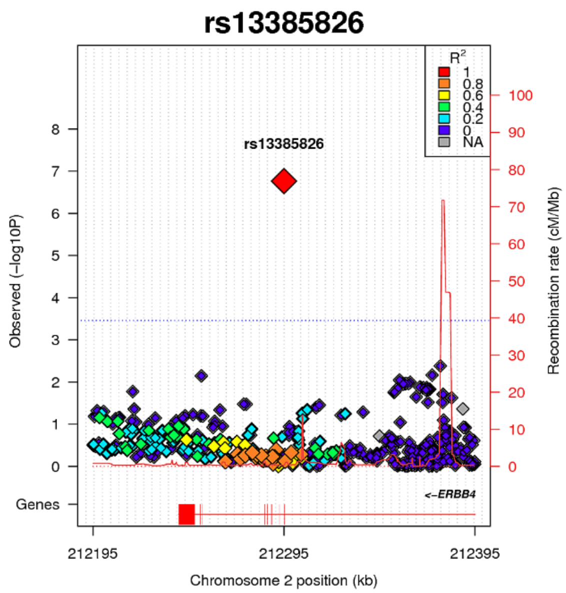

Supplement: Supplementary Figure 3 [file tp2017183x3.pdf]
