# Supplementary material for: Neuregulin signaling pathway in smoking behavior
Source: Transl Psychiatry. 2017 Aug 22;7(8):e1212–. doi: 10.1038/tp.2017.183 (PMC5611747; doi:10.1038/tp.2017.183)

Supplementary figure 4. Regional plot for *NRG3* SNP rs11192578 showing association with NW symptom score. Threshold line corresponds to FDR p=0.05.

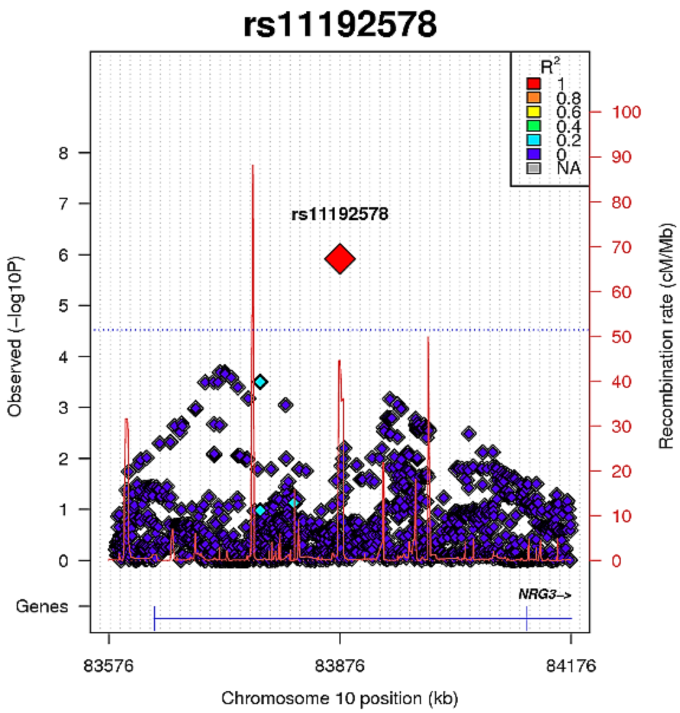

Supplement: Supplementary Figure 4 [file tp2017183x4.pdf]
