# Supplementary material for: Neuregulin signaling pathway in smoking behavior
Source: Transl Psychiatry. 2017 Aug 22;7(8):e1212–. doi: 10.1038/tp.2017.183 (PMC5611747; doi:10.1038/tp.2017.183)

Supplementary figure 5. Boxplot showing expression level difference between current daily, occasional and never smoker for *NRG1* and *PSEN1*.

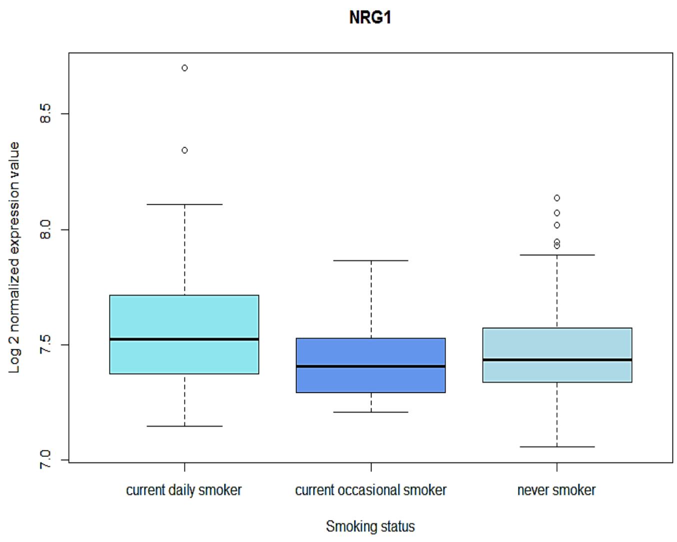

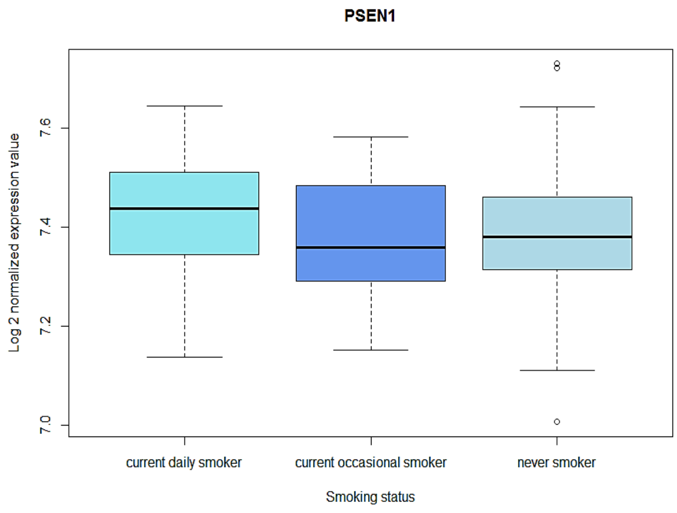

Supplement: Supplementary Figure 5 [file tp2017183x5.pdf]
